# Broken Tip of a Disposable Saliva Ejector as a Bronchial Foreign Body in a Patient With Severe Physical and Intellectual Disabilities

**DOI:** 10.7759/cureus.50854

**Published:** 2023-12-20

**Authors:** Daisuke Himeji, Ritsuya Shiiba, Gen-ichi Tanaka, Hidemi Shimonodan, Kotaro Kojima

**Affiliations:** 1 Department of Internal Medicine, Miyazaki Prefectural Miyazaki Hospital, Miyazaki, JPN; 2 Department of Pediatrics, Miyazaki Prefectural Miyazaki Hospital, Miyazaki, JPN

**Keywords:** foreign body aspiration, severe physical and intellectual disabilities, bronchoscopy, saliva ejector, bronchial foreign body

## Abstract

Oral care for patients with severe physical and intellectual disabilities is important to prevent the development of systemic diseases and maintain or improve their health. Foreign bodies accidentally aspirated into the respiratory tract can cause critical problems. To our knowledge, this is the first case report of aspiration of a broken tip of a disposable saliva ejector in a patient with severe physical and intellectual disabilities. The patient’s strong bite broke off the ejector’s tip during oral care. The foreign body was removed by flexible bronchoscopy without any complications. Such cases are sometimes asymptomatic or mildly symptomatic; thus, learning how to appropriately respond is essential for caregivers and family doctors. In addition, this device is widely used in clinical practice, and such risks should be widely known. Moreover, manufacturers should develop more robust equipment for oral care.

## Introduction

Oral care for a patient with severe physical and intellectual disabilities is important for daily oral hygiene, prevention of systemic diseases, and maintenance of improvement of health [1], which improves the patient’s quality of life. However, in the oral care of these patients, accidental aspiration of small foreign bodies into the respiratory tract can sometimes become a critical problem [2].

Devices such as suction catheters are widely used in clinical practice for intraoral suction. There are a couple of reports of damaged suction catheters resulting in bronchial foreign bodies during intratracheal suction [3], but there are no reports of bronchial foreign bodies caused by damaged suction devices during intraoral suction. Among those suction devices, a saliva ejector is a tubular device that provides suction to remove saliva and debris from the mouth of dental and critically ill patients to maintain a clear operative field or to clean the oral cavity [4]. We report a case of aspiration of a broken saliva ejector tip as a bronchial foreign body in a patient with severe physical and intellectual disabilities. To our knowledge, this is the first report of a case of a bronchial foreign body aspiration caused by the use of a broken saliva ejector.

## Case presentation

A 31-year-old man with severe physical and intellectual disabilities was admitted to the Miyazaki Prefectural Miyazaki Hospital due to suspicion of an aspiration of a bronchial foreign body. The patient had symptomatic epilepsy, severe mental disabilities, and spastic quadriplegia.

Three days before visiting our hospital, the patient’s mother had used a saliva ejector to suction his oral cavity at home. She subsequently noticed that the tip of the saliva ejector was missing, so she consulted their family doctor. Chest radiography and gastrointestinal endoscopy were performed to determine if the suspected bronchial foreign body was present, but it was not found. The doctor instructed his mother to observe his condition. His mother thought that it was strange that the amount of the patient’s sputum increased thereafter. The day before the visit to our hospital, the patient’s SpO_2_ was low and the possibility of a bronchial foreign body aspiration was considered. The patient was then referred to our department.

During the admission, his vital signs were as follows: body temperature, 37.2°C; blood pressure, 114/90 mmHg; heart rate, 115 beats/minute, and respiratory rate, 26 cycles/minute, with an oxygen saturation of 88%. Chest auscultation revealed decreased breath sounds in the left lung field and bilateral rhonchi.

**Figure 1:**
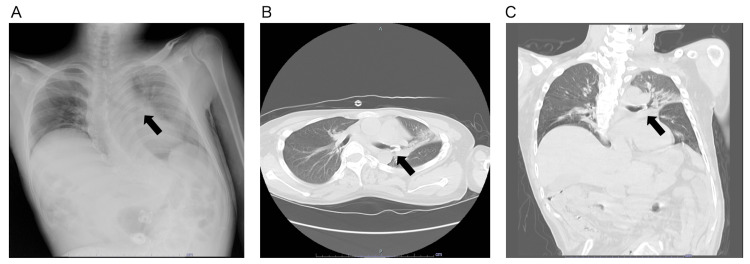
Chest radiograph and computed tomography (CT) images obtained during admission. A. A chest radiograph showing a foreign body (black arrow) in the left upper bronchus. B. Axial CT showing a U-shaped plastic foreign body in the left upper bronchus (black arrow). C. Coronal CT showing a U-shaped plastic foreign body in the left upper bronchus (black arrow).

His hemogram revealed the following findings: white blood cell count, 13,180 cells/mm^3^ (59.3% neutrophils, 32.8% lymphocytes, 5.2% monocytes, 1.9% eosinophils, and 0.8% basophils); and C-reactive protein level, 0.47 mg/dL. Chest radiography showed decreased permeability of the left lung and a foreign body in the left upper lobar bronchus (Figure 1A). A foreign body in the left upper lobar bronchus was seen in the subsequent chest CT (Figures 1B, 1C).

**Figure 2:**
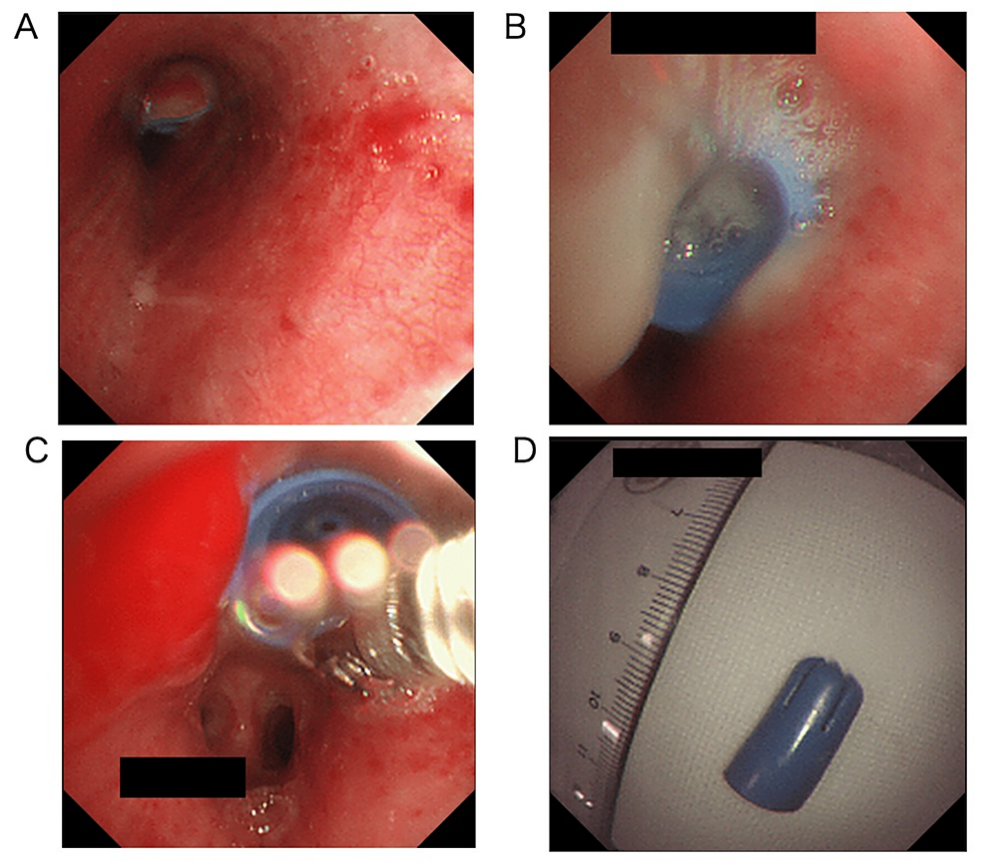
Endoscopic view of the foreign body. A. Distal endoscopic view of the foreign body in the left upper bronchus. B. Proximal endoscopic view of the foreign body. C. Removal of the foreign body using grasping forceps. D. Removed broken tip of the saliva ejector.

The patient underwent emergent flexible bronchoscopy under local anesthesia and deep sedation using midazolam and fentanyl. Copious oral secretions were noted, and the patient was moving vigorously and clenching the mouthpiece with great force. A foreign body shaped like a saliva ejector’s tip was noted in the left upper bronchus, which obstructed the upper lobe bronchus of the left lung (Figures 2A, 2B). We performed endotracheal intubation (inner diameter 8 mm) on the patient and removed the foreign body en bloc with grasping forceps (FB-32C-1, Olympus, Tokyo, Japan) (Figure 2C). The procedure was completed successfully without any complications. The foreign body was identified as a saliva ejector tip (Figure 2D).

After the endoscopic procedure, 125 mg methylprednisolone was administered intravenously to prevent airway edema. Moreover, as purulent sputum was found in the bronchi, the patient was suspected of having pneumonia. Antibiotics (sulbactam/ampicillin) were administered for one week, and he was discharged on postoperative day eight.

## Discussion

We reported a case of aspiration of a broken tip of a disposable saliva ejector as a bronchial foreign body in a patient with severe physical and intellectual disabilities. This appears to be the first report of such a case. As airway foreign bodies are life-threatening, it is critical to prevent such occurrences and for medical professionals to respond appropriately when they occur.

**Figure 3:**
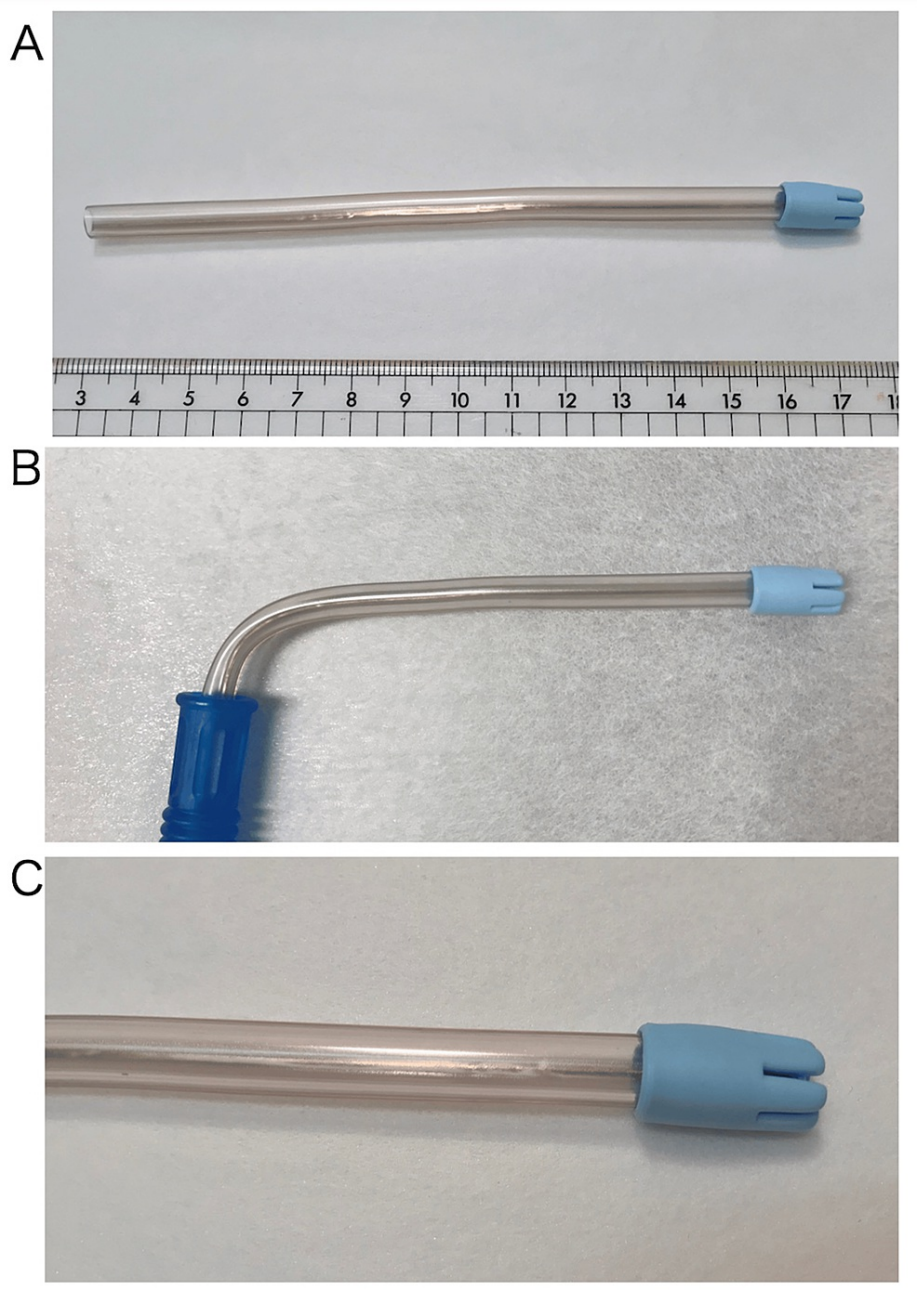
A disposable saliva ejector. A. Distant view. B. The ejector usually has a J-shaped appearance and comprises an inlet (longer arm of the J) placed in the oral cavity and an outlet (stem of the J) connected to a vacuum source via a flexible hose. C. Proximal view of the disposable saliva ejector’s tip.

People with physical or intellectual disabilities require more care and supervision in all activities of daily living, including those related to their oral health care [1]. Various devices are used for oral care, including a disposable saliva ejector (Premium Plus Japan, Osaka, Japan), which is a tube-like device with a rubber cover at the tip to prevent damage to the oral cavity (Figure 3A) [4]. This device is used in the oral care of many types of inpatients, not just those with disabilities such as our patient. Before its use, the saliva ejector has a J-shaped appearance and comprises an inlet (the longer arm of the J) placed in the mouth cavity and an outlet (the stem of the J) connected to a vacuum source via a flexible hose (Figure 3B). The saliva ejector tip is firmly attached to the plastic tube and has a structure that prevents it from easily coming off (Figure 3C).

The mother of our patient confirmed that there were no problems with the tip, as she checked the device in advance before its use. She reported that because of his cerebral palsy, her son bit the device hard, sometimes very firmly, when she was doing oral care. The saliva ejector is unlikely to break under regular use, but it is crucial to be aware of the potential for breakage in some patients. Furthermore, care is needed not to damage the tube or tip before and during use. It would be helpful if the manufacturer(s) would further improve the device’s structure by integrating the tip into the whole tube.

The clinical manifestations of foreign body aspiration range from asymptomatic presentations to life-threatening emergencies. Generally, airway foreign bodies rarely cause acute choking symptoms and are often noticed by coughing, increased sputum production, and shortness of breath [5]. In the present case, the mother noticed an increased sputum volume, prompting imaging examinations and the eventual discovery of a foreign body in the respiratory tract. Severely disabled people are less able to complain of symptoms; thus, healthcare professionals need to know these signs, i.e., changes in breathing patterns and increased sputum production, which could indicate airway foreign bodies. Additionally, using chest radiography to identify radiolucent foreign bodies is challenging [6]. Compared with plain chest radiography, CT can help localize and evaluate the nature of a foreign body and could provide more accurate information. A foreign body is visible on CT in 62% of cases, which could help identify indicative consolidation, atelectasis, and bronchial wall abnormalities [5].

In our patient, the foreign body could not be identified in the initial chest radiography at their family doctor’s clinic for unknown reasons. Several groups have proposed charts for detecting foreign bodies in the airways, and it is vital to use them to detect foreign bodies at an early stage [2,7].

Generally, bronchial foreign bodies often enter the right bronchus because of the bronchial structures. In this case, the foreign body was located in the left upper lobe branch. The patient had right-sided curvature due to the underlying disease. As a result, the carina was displaced to the left anterior surface of the vertebral body, and the right main bronchus was slightly compressed between the anterior chest wall and the vertebral body. Additionally, the patient tended to prefer the left lateral position. These are likely the reasons why the foreign body entered the left bronchus. In this way, when estimating the location of a bronchial foreign body in a physically and intellectually disabled person, it is necessary to take anatomical factors into consideration.

The rigid bronchoscope is still considered the safest instrument in most respiratory centers for foreign body removal, although flexible bronchoscopy seems to be the preferred method for treating adult airway problems [8]. Flexible bronchoscopes can remove almost any type of foreign body in adults because of the availability of several ancillary instruments, including forceps or a balloon. Thus, in adults with airway foreign body aspiration, flexible bronchoscopy is routinely performed as an initial assessment at our institution. In airway foreign body removal, it is crucial to consider the foreign body’s expected morphology, choose various forceps and other appropriate devices, and prepare for possible adverse events, including bleeding and respiratory failure.

## Conclusions

This seems to be the first report of a bronchial foreign body caused by a broken tip of a disposable saliva ejector in a patient with severe physical and intellectual disabilities. This device is widely used in clinical practice, and such risks should be widely known. In addition, it is pertinent to develop devices that are not easily damaged and educate family members and doctors about the symptoms of airway foreign bodies and ways to manage them.

## References

[1] Furuta M, Yamashita Y (2013). Oral health and swallowing problems. Curr Phys Med Rehabil Rep.

[2] Yadav RK, Yadav HK, Chandra A, Yadav S, Verma P, Shakya VK (2015). Accidental aspiration/ingestion of foreign bodies in dentistry: a clinical and legal perspective. Natl J Maxillofac Surg.

[3] Blohm ME, Vezyroglou K, Riedel F, Roth B, Singer D (2011). Suction catheter tip as an endobronchial foreign body. Intensive Care Med.

[4] Lin LW, Chong CF (2021). Saliva ejector assisted laryngoscopy (SEAL) for protective intubation. Am J Emerg Med.

[5] Ng J, Kim S, Chang B, Lee K, Um SW, Kim H, Jeong BH (2019). Clinical features and treatment outcomes of airway foreign body aspiration in adults. J Thorac Dis.

[6] Bajaj D, Sachdeva A, Deepak D (2021). Foreign body aspiration. J Thorac Dis.

[7] Hitter A, Hullo E, Durand C, Righini CA (2011). Diagnostic value of various investigations in children with suspected foreign body aspiration: review. Eur Ann Otorhinolaryngol Head Neck Dis.

[8] Blanco Ramos M, Botana-Rial M, García-Fontán E, Fernández-Villar A, Gallas Torreira M (2016). Update in the extraction of airway foreign bodies in adults. J Thorac Dis.

